# Identification of *RNPC3* as a novel *JAK2* fusion partner gene in B‐acute lymphoblastic leukemia refractory to combination therapy including ruxolitinib

**DOI:** 10.1002/mgg3.1110

**Published:** 2019-12-30

**Authors:** Xue Chen, Fang Wang, Yang Zhang, Xiaoli Ma, Mingyue Liu, Panxiang Cao, Lin Zhou, Lan Wang, Xian Zhang, Tong Wang, Hongxing Liu

**Affiliations:** ^1^ Divison of Pathology & Laboratory Medicine Hebei Yanda Lu Daopei Hospital Langfang China; ^2^ Department of Hematology Hebei Yanda Lu Daopei Hospital Langfang China; ^3^ Divison of Pathology & Laboratory Medicine Beijing Lu Daopei Hospital Beijing China; ^4^ Beijing Lu Daopei Institute of Hematology Beijing China

**Keywords:** B‐acute lymphoblastic leukemia, *JAK2*, *RNPC3*, *RNPC3‐JAK2*

## Abstract

**Background:**

Hematopoietic neoplasms with chromosomal translocations involving *JAK2* are rare, and most of them show myeloproliferative neoplasm‐associated features, followed by B‐acute lymphoblastic leukemia (B‐ALL). De novo B‐ALL cases with *JAK2* rearrangements are suggested to be appropriately considered as *BCR‐ABL1*‐like B‐ALL, but its partners varied.

**Methods:**

Fluorescence in situ hybridization (FISH), RNA sequencing (RNA‐Seq), whole‐genome sequencing, and reverse transcription polymerase chain reaction (RT‐PCR) were performed to identify the pathogenic fusion gene in a 29‐year‐old woman with relapsed B‐ALL and rare t(1;9)(p13;p22) translocation.

**Results:**

We identified *RNPC3* as a new *JAK2* fusion partner in the patient. She was treated with a combination of chemotherapy and targeted drug ruxolitinib and chimeric antigen receptor T‐cell therapy, but failed to achieve complete remission. She had no chance to undergo allogeneic hematopoietic stem cell transplantation and died of disease progression 7 months after the initial diagnosis. Her clinical course demonstrated that this novel *RNPC3‐JAK2* fusion might portend an unfavorable prognosis.

**Conclusion:**

This finding adds to the expanding compendium of *JAK2* fusions found in B‐ALL and suggests the potential need for a diagnostic FISH analysis as well as RNA‐Seq in the appropriate clinical setting.

## Graphical abstract

We identify RNPC3 as a new JAK2 fusion partner in a 29‐year‐old female with relapsed B‐ALL by fluorescence in situ hybridization (FISH), RNA sequencing (RNA‐Seq), whole‐genome sequencing, and RT‐PCR. Her clinical course demonstrated that this JAK2 rearrangement might portend an unfavorable prognosis. This finding adds to the expanding compendium of JAK2 rearrangements found in B‐ALL and suggests the potential need for a diagnostic FISH analysis as well as RNA‐Seq in the appropriate clinical setting.

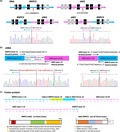

## 1 INTRODUCTION

The *JAK2* gene (OMIM#147796; NM_004972.3), which locates on 9p24, encodes a cytoplasmic tyrosine kinase belonging to the Janus kinase family that activates multiple downstream transducers through the JAK‐STAT pathway and plays an essential role in hematopoiesis, cell proliferation, and differentiation (Tang et al., 2019). Gain of function mutations, translocations, and amplifications involving the *JAK2* gene and leading to constitutive activation of the JAK2 kinase have been reported in various hematological malignancies. The most common mutation is V617F, which leads to constitutive activation of JAK2 and has been reported in more than 95% cases of polycythemia vera and approximately 50%–60% cases of essential thrombocythemia and primary myelofibrosis (Arber et al., 2016). Amplifications of *JAK2*, although rare, are described mainly in primary mediastinal large B‐cell lymphoma and Hodgkin's lymphoma (Patnaik et al., 2010).

Hematopoietic neoplasms with chromosomal translocations involving *JAK2* are rare, and most of them show myeloproliferative neoplasm (MPN)‐associated features, often with eosinophilia. Patients with *PCM1‐JAK2* fusion can be diagnosed as “myeloid/lymphoid neoplasms with *PCM1‐JAK2*”, which is now incorporated into the 2016 revision of WHO classification of neoplastic diseases of the hematopoietic and lymphoid tissues as a provisional entity (Arber et al., 2016). Cases with translocations resulting in *JAK2* fusions may also present with B‐lymphoblastic leukemia (B‐ALL) and have the features of *BCR‐ABL1*‐like B‐ALL. Up to date, at least 20 different partner genes of *JAK2* have been identified in *BCR‐ABL1*‐like B‐ALL (Tang et al., 2019). The most common of them include *PAX5*, *SSBP2*, *BCR*, *ATF7IP*, and *ETV6* (Gu et al., 2019; Li et al., 2018; Reshmi et al., 2017; Roberts et al., 2017, 2014).

The identification of *JAK2* alterations is considered to be beneficial for the treatment of patients because of the possibility of response to *JAK2* inhibitors. Here, we report the identification of a novel *RNPC3‐JAK2* fusion in a refractory case of B‐ALL with rare t(1;9)(p13;p22) translocation.

## 2 MATERIALS AND METHODS

### 2.1 Ethical compliance

The study was approved by the Institutional Review Board and Ethical Committee of the Hebei Yanda Lu Daopei Hospital. Informed consent was obtained from the patient. Samples were obtained following the Declaration of Helsinki and the Chinese legislation for the protection of personal data and research on human samples.

### 2.2 Subject

A case of relapsed, refractory B‐ALL patient with rare t(1;9)(p13;p22) translocation was selected for further investigation. Peripheral blood (PB) and bone marrow (BM) samples of the patient were collected for routine diagnosis and archived for further research.

### 2.3 Cytogenetic and molecular cytogenetic analysis

BM samples at relapse were processed after short‐term (24 hr) and unstimulated culture following standard G banding procedures. Twenty metaphases were analyzed, and results were reported according to the 2016 International System for Human Cytogenetic Nomenclature (ISCN 2016) recommendations. Fluorescence in situ hybridization (FISH) analysis was performed on 500 interphase cells using the Vysis LSI *JAK2* dual‐color break‐apart probe (Abbott Laboratories) according to the manufacture's recommendations.

### 2.4 RNA sequencing (RNA‐Seq) and fusion validation

RNA‐Seq was performed using RNA extracted from the BM sample by HiSeq 2,500 (Illumina Inc.) to search for the potential fusion gene. Reads were mapped and processed by combining Arriba (v1.0.1; Hunt et al., 2017) and STAR‐Fusion (v1.3.1; Haas et al., 2017) to analyze the gene fusions. For the validation of the RNA‐Seq results, reverse transcription polymerase chain reaction (RT‐PCR) was performed followed by Sanger sequencing.

### 2.5 Whole‐genome sequencing (WGS)

On the genomic DNA of BM sample 30 × WGS was performed using HiSeq X Ten (Illumina, Inc.). Raw reads in fastq were preprocessed and controlled for quality using fastp (Chen, Zhou, Chen, & Gu, 2018; Chen, Wang, et al., 2018), followed by rapid genome analysis using speedseq (Chiang et al., 2015) with default parameters. Structural variants were called from speedseq with default options, and the next annotation tool was AnnotSV (Geoffroy et al., 2018) with various databases to filter the pathogenic/likely pathogenic somatic copy number variants (CNVs).

### 2.6 Targeted next‐generation sequencing and mutation analysis

Mutation hotspots or whole coding regions of 86 genes that are known to be frequently mutated in hematologic malignancies were sequenced using a targeted, multiplexed amplicon‐based high‐throughput sequencing protocol as we previously reported (Zhang et al., 2018).

## 3 RESULTS

### 3.1 Case report

The 29‐year‐old female was initially admitted to a local hospital because of a persistent fever without an obvious cause. Blood tests showed a white blood cell count of 25.46 × 10^9^/L, hemoglobin level of 87 g/L, and platelet count of 39 × 10^9^/L. Morphologic examination of BM smears disclosed infiltration by 94% of lymphoblasts. Flow cytometry (FCM) revealed 90.5% of blast cells expressing B‐lymphoid–related markers and CD123. She was diagnosed as B‐ALL and immediately started an induction chemotherapy regimen that consisted of cyclophosphamide, vindesine, idarubicin, prednisone, and pegaspargase. A lumbar puncture at the time of diagnosis revealed no involvement of the cerebrospinal fluid. She achieved complete remission (CR) after a course of chemotherapy but then developed central nervous system leukemia with 69.2% blasts in cerebrospinal fluid.

**Figure 1:**
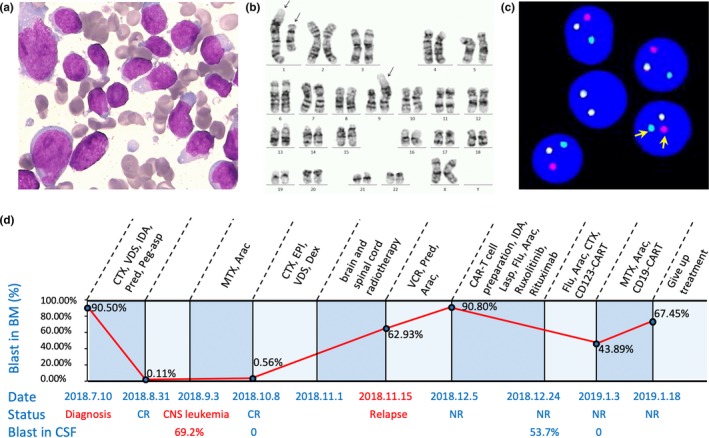
Morphology, karyotyping, FISH analysis, and clinical course. (a) Wright‐Giemsa stained bone marrow (BM) smear at relapse, showing infiltration of lymphoblasts. (b) G‐banding karyotype of the BM sample at relapse showing 46, XX, dup(1)(q21q42), t(1;9)(p13;p22). The arrows indicate duplicated chromosome 1, translocated chromosomes 1 and 9, respectively. (c) Representative interphase nuclei in the relapsed BM sample demonstrating disruption of the *JAK2* probe. A normal result will demonstrate an intact red/green fusion signal, whereas an abnormal result will demonstrate the separation of the red and green signals as indicated by the arrows. (d) Clinical timeline of the patient's treatment history from diagnosis to the end of treatment. Treatment at various time points is shown along the top of the timeline; the red line indicates the percentage of blast cells in BM monitored by flow cytometry; the clinical response is indicated along the bottom; the percentage of blast cells in CSF detected by flow cytometry is also shown along the bottom. Arac, cytarabine; CR, complete remission; CTX, cyclophosphamide; Dex, dexamethasone; EPI, epirubicin; Flu, Fludarabine; IDA, idarubicin; IDA, idarubicin; Lasp, L‐asparaginase; MTX, methotrexate; NR, no remission; Peg‐asp, pegaspargase; Pred, prednisone; VCR, vincristine; VDS, vindesine

After another two courses of chemotherapy, she received brain and spinal cord radiotherapy for the preparation of allogeneic hematopoietic stem cell transplantation (allo‐HSCT). However, 2 days after the end of radiotherapy, a rapid systemic relapse occurred, and the patient was admitted to our hospital. BM smears showed 92.5% of lymphoblasts (Figure 1a), and FCM revealed 62.93% of B lymphoblasts in BM. Chromosome karyotyping showed t(1;9)(p13;p22) translocation (Figure 1b), and multiplex‐nested RT‐PCR designed to amplify 36 fusion genes was negative (Chen, Wang, et al., 2018; Chen et al., 2019). CR was failed to achieve again, although multiple attempts, including the combination of chemotherapy and chimeric antigen receptor (CAR) T‐cell therapy, were made (Figure 1d). The patient gave up treatment and died of disease progression 7 months after the initial diagnosis.

### 3.2 Detection of *JAK2* rearrangement by FISH analysis

The FISH analysis was performed on metaphase chromosomes using *JAK2* dual‐color break apart probe and detected split signals in 73% of interphase nuclei (Figure 1c) suggests an involvement of the cleavage and rearrangement of JAK2 gene due to t(1;9)(p13;p22) translocation.

### 3.3 Identification of *JAK2* fusion transcript by RNA‐Seq

**Figure 2:**
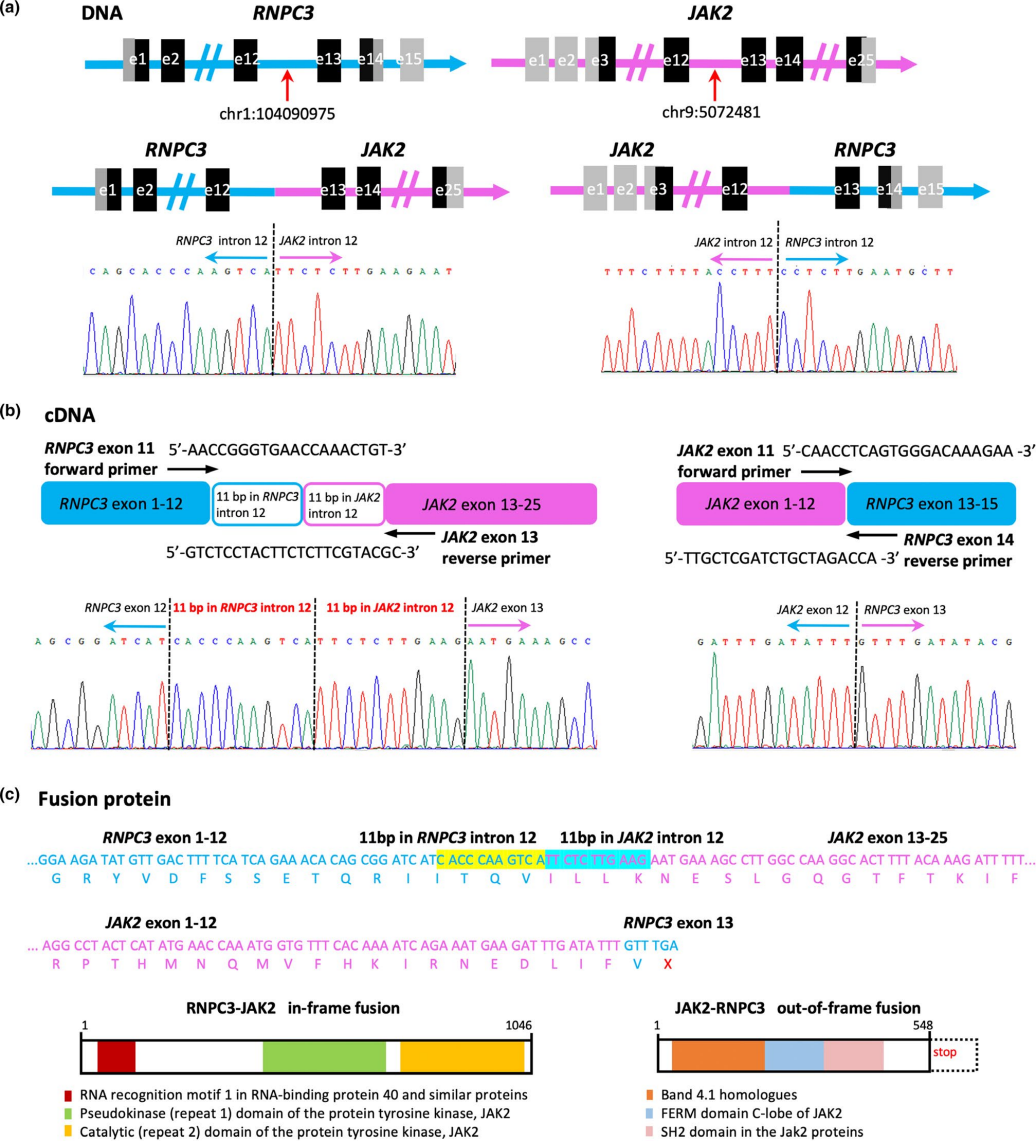
Identification of the *RNPC3‐JAK2* in‐frame fusion and the *JAK2‐RNPC3* out‐of‐frame fusion. (a) WGS analysis revealed breakpoints in intron 12 of *RNPC3* and intron 12 of *JAK2*, respectively. The genomic splicing sequences were validated by PCR and Sanger sequencing. (b) RT‐PCR using primers in *RNPC3* exon 11 and *JAK2* exon 13 and Sanger sequencing of the PCR product revealed an atypical *RNPC3* exon11 to *JAK2* exon 13 fusion with a 22‐bp exonized sequence. RT‐PCR followed by direct sequencing also confirmed the *JAK2* exon 12 to *RNPC3* exon 13 fusion. (c) The 22‐bp exonized sequences permit the *RNPC3‐JAK2* fusion an in‐frame one, which encoded 1,046 amino acids. The RNPC3‐JAK2 fusion protein preserved both the JAK2 pseudo‐kinase domain and the catalytic domain. The predicted structure of the JAK2‐RNPC3 fusion protein contained 547 amino acids, which were encoded by *JAK2* and one amino acid encoded by *RNPC3*

RNA‐Seq performed on the patient's BM sample collected at recurrence showed an out‐of‐frame fusion of *JAK2* exon 12 to *RNPC3* (OMIM#618016; NM_017619.4) exon 13. RT‐PCR followed by Sanger sequencing confirmed the fusion transcript (Figure 2b). The predicted structure of the *JAK2‐RNPC3* fusion protein contained 547 amino acids, which were encoded by *JAK2* and one amino acid encoded by *RNPC3* (Figure 2c). The fusion protein can be deemed as a truncated form of JAK2 protein with a COOH‐terminal deletion of the pseudokinase and catalytic domains of the JAK2 protein tyrosine kinase, which is unlikely to be the critical pathogenic fusion for leukemia pathogenesis.

### 3.4 Identification of genomic breakpoints

WGS analysis was performed on DNA isolated from the patient's relapsed BM sample to clarify the genomic breakpoints in *JAK2* and *RNPC3* genes. Results revealed the breakpoints in *JAK2* intron 12 and *RNPC3* intron 12, respectively. The genomic fusion sequences were also validated by PCR and Sanger sequencing (Figure 2a).

### 3.5 Identification of the *RNPC3‐JAK2* transcript

In order to seek for the possible reciprocal *RNPC3‐JAK2* fusion transcript, RT‐PCR was performed using primers in *RNPC3* exon 11 and *JAK2* exon 13. Sanger sequencing of the PCR product revealed an atypical fusion of *RNPC3* exon12 to *JAK2* exon 13, with two 11‐bp exonized sequences insertion which derived from the adjacent *RNPC3* intron 12 and *JAK2* intron 12, respectively (Figure 2b). The 22‐bp exonized sequence permits the fusion transcript an in‐frame one, which encoded 1,046 amino acids. The fusion protein preserved the tyrosine kinase domain of JAK2, thus would contribute to constitutive activation of the JAK‐STAT pathway (Figure 2c).

### 3.6 Inversion of the *PAX5* gene

**Figure 3:**
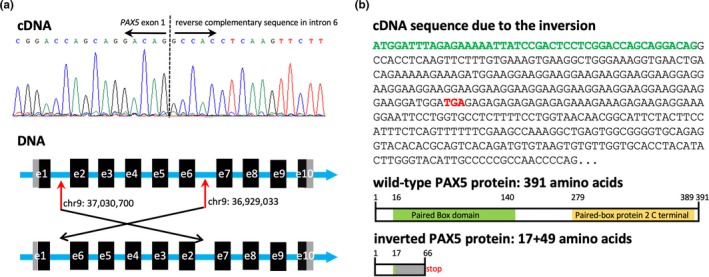
Intragenic inversion of the *PAX5* gene. (a) RNA‐Seq showed *PAX5* exon 1 was fused to the reverse complementary sequence in intron 6, which was validated by RT‐PCR and Sanger sequencing. WGS analysis revealed breakpoints in *PAX5* intron 1 and intron 6, respectively. (b) The internal focal inversion resulted in a frameshift and malfunction coding protein of 66 amino acids, including 49 weltered C terminal amino acids compared to the wild‐type PAX5 protein

RNA‐Seq also showed an intragenic inversion of the *PAX5* gene. *PAX5* exon 1 was fused to the reverse complementary sequence in intron 6, which was validated by RT‐PCR and Sanger sequencing (Figure 3a). WGS analysis revealed breakpoints in *PAX5* intron 1 and intron 6, respectively. The internal focal inversion resulted in a frameshift and a malfunction coding protein of 66 amino acids, including 49 weltered C terminal amino acids compared to the wild‐type PAX5 protein (Figure 3b).

### 3.7 Mutation analysis of 86 genes

*NRAS* Q61H mutation and *PAX5* G367S mutations were identified in the patient's relapsed BM sample, both with a variant allele frequency of 41%.

### 3.8 Genome CNVs analysis

Deletions of *IKZF1*, *CDKN2A*, *BTG1*, *CDK6*, *ADARB2*, *ETV6*, and *CREBBP* were detected in the patients’ relapsed BM sample by WGS and structural variation analysis.

## 4 DISCUSSION

The *RNPC3* gene, which locates at 1p21.1, was first cloned and characterized in 2003 from a human fetal brain cDNA library. It encodes a protein with two RNA recognition motifs (RRMs), which functions in important steps of posttranscriptional regulation of gene expression and is involved in the processing and transport of mRNA precursors (Zhao et al., 2003). Splicing is an essential step in eukaryotic gene expression. Two types of spliceosomes catalyze the process of pre‐mRNA splicing: the major U2‐type spliceosome is found in all eukaryotes and removes U2‐type introns, which represent more than 99% of pre‐mRNA introns; the minor U12‐type spliceosome is found in some eukaryotes and removes U12‐type introns, which are rare and have distinct splice consensus signals. The 65 kDa protein encoded by *RNPC3* is a component of the U12‐type spliceosome (Doggett et al., 2018).

Little is known about the function of RNPC3 protein in human cancer, and no *RNPC3* gene abnormality has ever been reported in hematological malignancies. Our investigation provides the first reported example of *RNPC3* rearrangement in lymphoid neoplasm.

The JAK2 protein has two crucial domains: a catalytic domain (JH1) and a pseudokinase domain (JH2). JH1 is the active tyrosine kinase domain and possesses the tyrosine residues, which are phosphorylated when JAK2 is activated. JH2 is a kinase‐like domain lacking actual kinase activity and exerts an inhibitory effect on JH1 (Smith & Fan, 2008). The FERM homology domains, which are located near the amino terminus of the JAK2 protein, are responsible for the noncovalent binding to the juxta‐membrane cytoplasmic region of the type I cytokine receptors. When a ligand binds a type I cytokine receptor, a conformational change in the receptor brings two JAK2 proteins close enough together to allow them to phosphorylate each other. Phosphorylated JAK2 acts as an activated tyrosine kinase and further activates multiple downstream transducers through the JAK‐STAT pathway. Translocations of *JAK2*, however, provide a dimerization interface to the JAK2 kinase domain by the fusion partners and induce the dimerization or oligomerization of JAK2 without ligand binding, which also results in constitutive activation of JAK2 (Smith & Fan, 2008).

The breakpoints in *JAK2* and its partner genes were variable in reported cases with *JAK2* fusions, without apparent disease correlation. Despite these inconsistencies, all *JAK2* fusions preserve its JH1 domain with or without JH2 domain. Moreover, the fusion partners of *JAK2* contain domains which can facilitate dimerization of JAK2 protein molecule and subsequently cause constitutive kinase activation (Kawamura, Taki, Kaku, Ohki, & Hayashi, 2015; Poitras, Cin, Aster, Deangelo, & Morton, 2008; Roberts et al., 2012; Yano et al., 2015). The formation of alternative splice sites in introns of *RNPC3* and *JAK2* genes and the additional 22‐bp exonized sequence permits the fusion transcript in‐frame and retains both the JH2 and JH1 coding sequences in this case. The preservation of *JAK2* from exon 13 was reported in one case with *OFD1‐JAK2* fusion (Yano et al., 2015). The RNPC3 protein is predicted to contain two coiled‐coil motifs (Coils v2.1; http://www.ch.embnet.org/software/COILS_form.html), and both of which are retained in the RNPC3‐JAK2 fusion protein. As has been found for other tyrosine kinase fusion proteins in leukemia, the coiled‐coil motifs derived from RNPC3 likely contribute to the dimerization or oligomerization of RNPC3‐JAK2 chimera, with consequent constitutive activation of the JAK2 kinase domain.

An *PAX5* internal focal inversion, which results in a frameshift and malfunction coding protein, was also identified in this case. Although this inversion has not been reported before, *PAX5* alterations resulting in a truncated form of proteins have been reported to behave as competitive inhibitors of wild‐type PAX5 transactivating activity (Coyaud et al., 2010). Furthermore, somatic mutations of *NRAS* Q61H and *PAX5* G367S, as well as deletions of *IKZF1*, *CDKN2A*, *BTG1*, *CDK6*, *ADARB2*, *ETV6*, and *CREBBP* were also detected. The *RNPC3‐JAK2* fusion is likely acquired early in leukemogenesis and drive transcriptional and epigenetic dysregulation and aberrant self‐renewal. Additional lesions and secondary genetic alterations might contribute to lymphoid development disruption and maturation arrest (Mullighan, 2012).

B‐ALL was the second‐most common presentation among patients with *JAK2* fusions (Tang et al., 2019). In cases without a documented MPN and lack of eosinophilia, such cases are suggested to be appropriately considered as *BCR‐ABL1*‐like B‐ALL, which is characterized by a gene‐expression profile similar to that of *BCR‐ABL1*‐positive ALL, alterations of lymphoid transcription factor genes, and a poor outcome (Roberts et al., 2014). The reported B‐ALL patients with *JAK2* fusions often presented with hyperleukocytosis and responded unfavorably to chemotherapy with frequent relapse, and about one third of patients died from the disease during 33 months of follow‐up (Tirado et al., 2010). Our case failed to achieve CR again after the first relapse, although multiple attempts were made, including the combination of chemotherapy and targeted drug ruxolitinib and CAR‐T cell therapy. The clinical course suggested that this novel *RNPC3*‐*JAK2* fusion also portend an unfavorable prognosis. Unfortunately, the molecular pathogenesis of the patient was not elucidated when she was initially diagnosed in the local hospital, and ruxolitinib was not applied timely.

As RNA‐Seq is increasingly used in clinical practice, more cases with *JAK2* fusions might be identified timely and effectively, especially those with variant partner genes. Only the reciprocal *JAK2‐RNPC3* but not the chief culprit *RNPC3‐JAK2* transcript was identified directly by RNA‐seq in this case, which is mainly due to the adverse penalty contributed by the 22‐bp exonized insertion in sequence alignment during the process of bioinformatics analysis. The necessity of additional insertion sequences to maintain the reading frame might be the essential reason for the rare occurrence of such fusion genes.

We deciphered a novel pathogenetic *RNPC3‐JAK2* fusion in an adult B‐ALL patient with rare t(1;9)(p13;p22) translocation. Integrated genomic analysis plays an important role in elucidating the critical molecular mechanisms of pathogenesis. The identification of the new partner gene of *JAK2* helps in characterizing of B‐ALL and in monitoring minimal residual disease after treatment. Also, cases with this fusion may benefit from *JAK2* inhibitors, such as ruxolitinib. Further investigation is worthy of being conducted to elucidate the oncogenic properties of *RNPC3‐JAK2* and confirm the clinical and biological features of patients harboring this fusion gene.

## CONFLICT OF INTEREST

The authors declare that they have no conflict of interest.

## AUTHOR CONTRIBUTION

H.L. designed the research; X.C. designed molecular studies and wrote the study; F.W. and Y.Z. supervised clinical and experimental findings; X.M., M.L., L.Z., and L.W. performed molecular studies; P.C. performed bioinformatics analysis; X.Z. was involved in the management of the patient and provided clinical data. T.W. performed cytogenetic analysis. All authors reviewed the manuscript and contributed to the final draft.
